# Tuberculosis of the Colon

**Published:** 1909-09

**Authors:** Ernest W. Hey Groves

**Affiliations:** Assistant Surgeon, Bristol General Hospital


### TUBERCULOSIS OF THE COLON.

BY

Ernest W. Hey Groves, M.S., B.Sc., F.R.C.S.,
*Assistant Surgeon, Bristol General Hospital.*

Although the colon shows, with all other tissues of the body, a liability to invasion by both miliary and caseous tubercle, it has also a peculiar form of reaction, so unlike ordinary tuberculous manifestations that it has until recently been confounded with carcinoma. In 1891, however, Hartmann, in France, and Billroth, at Vienna, drew attention to the occurrence of large, hard, nodular masses in the cæcum which externally resembled cancer, but structurally were composed of a round cell and fibrous mass round tuberculous giant cell systems. The resemblance is made much more conspicuous by the similarity of clinical symptoms as this form of tuberculous disease causes slow intestinal obstruction rather than diarrhœa or ascites.

That the cæcum has a special disposition towards tuberculous infection is shown by the fact that of all cases of intestinal tuberculosis it is affected in 85 per cent. It alone is affected in about 9 per cent. And in the special forms of tuberculosis which we are now considering the region of the ilio-cæcal valve appears always to be the starting point of the disease, which may, nevertheless, spread far down the large intestine, even to the rectum itself (case 2).

Hartmann distinguishes two forms of pericæcal tuberculosis—the entero-peritoneal and the hyperplastic.

(1) *The entero-peritoneal form* attacks the mucous, submucous and serous coats of the bowel, producing ulceration and caseous masses. There is, however, but little fibrous tissue formed, and consequently there is no tendency to the formation of strictures. It often starts in the terminal part of the ileum, and spreads thence through the ileo-cæcal valve. The affection of the peritoneal coat causes an adhesive matting together of the bowel or the formation of a perforation. In this way peritonitis, local or diffuse, and fistulæ are common. Such a condition will be very liable to be mistaken for appendicitis, and in one of my cases (F. B.) the nature of the disease was not recognised until months after its onset, when the abdomen had to be opened to short circuit a fæcal fistula.

(2) *Hyperplastic form.*—This variety seldom attacks the ileum, but starting at or in the neighbourhood of the ileo-cæcal valve it first produces great induration of the cæcum and the adjacent part of the colon. In the majority of cases the disease spreads no further, but on the other hand it may either steadily advance down the colon or it may be deposited at intervals, with long stretches of healthy bowel between the lesions. Thus the hepatic flexure, the transverse colon, or the pelvic colon may each or all be the seat of induration, and in the case of a man of 35 (F. L.), recently under my care, the upper part of the rectum was similarly involved.

*Structure of the growth.*—The cæcum, which generally forms a large hard tumour, is often surrounded by a fibro-adipose mass, three or four cm. thick, similar to that which envelops many a chronic suppurating kidney. The associated lymph glands in front and behind the cæcum, together with those along the ileo-colic vessels, are enlarged—this enlargement occurs earlier and is more marked than in cases of carcinoma. Dobson and Jamieson, after excising an ileo-cæcal tumour, thought at the time to be cancerous, removed glands right up to the transverse part of the duodenum, and these, as well as the tumour, were proved microscopically to be tuberculous. The wall of the gut is transformed into a dense, rigid, fibrous tube, the lumen of which is so reduced as barely to admit a finger, or even in some cases a catheter. The huge thickness of the cæcal wall gives the mass its firm consistency, and in the case of a woman of 27, from whom the cæcum was excised by Cumston, the cæcal wall was actually 7 cm. thick.

Early in the disease the ileo-cæcal valve is destroyed, and from its site there projects into the lumen of the gut a sprouting mass of polypoid vegetations. Later, the mucous membrane is destroyed by ulceration, and the cul-de-sac of the cæcum is obliterated by contraction. According to Hartmann, the relation of the appendix to the disease is different in its two varieties, for whereas in the entero-peritoneal form it is often affected, being sometimes the primary focus, in the hyperplastic variety it is never involved, but its lumen remains widely dilated, opening into the narrowed interior of the cæcum.

Microscopically the main mass of the affected gut consists of fibrous tissue in different stages of development. Tubercles in the form of giant cell systems are sparsely scattered in the sub-mucous and subserous tissues, but the wide tract of thickened wall between these consists of small round cells or mature fibrous tissue. In my case (F. L.), where portions of the indurated great omentum and the mass outside the pelvic colon and rectum were removed for microscopical examination, nothing could be found except fibrous tissue, but the subsequent history of the case clearly proves that the disease was tuberculous.

Further, the great mass of fibro-adipose tissue outside the gut and the polypoid granulations within its lumen are further evidences of the simple inflammatory reaction, neither of these tissues showing any evidence of tuberculous structure. And as the essentially tuberculous nature of the affection is so overshadowed by a fibrous tissue reaction, it is probable that a certain proportion of so-called simple fibrous strictures are really instances of hyperplastic tubercle. The determination of the real nature of these conditions is rendered all the more difficult because the tubercle bacilli are so scanty as to be difficult of detection, even by inoculation experiments. This was my experience with a very advanced case (F. L.). When I opened the abdomen the second time it was full of fluid, and the nodules covering the peritoneal surface of the bowels were unmistakeable tubercles. Nevertheless, when some of the fluid was injected into guinea pigs at the Lister Institute, a negative result was obtained. Hartmann states definitely that in his cases inoculation experiments often give negative results, and in doubtful cases he says that the subsequent history of the patient gives a true clue to the nature of the disease. He refers to two cases as evidence of the tuberculous nature of apparently simple structures. One case died, ten years after excision, from phthisis, and the other, six years after excision, developed tuberculous peritonitis.

*Age and sex.*—The disease is most common between 20 and 40, and is decidedly rare before or after this period. But as instances of exceptional occurrence at the two extremes of life may be mentioned Guinon and Pater's case in a child of 4 and Cumston's case of a woman of 87. The fact that this peculiar form of tuberculous disease is generally found in the period of most vigorous adult life may possibly be related to the structure of the lesions. The disease, in its hyperplastic form especially, is a manifestation of a healthy reaction. In children and in old people, when the vitality does not permit of such tissue reaction, tuberculous disease will take the form of the ordinary ulceration of the bowel or miliary deposit on the peritoneum, where the tuberculous attack is met by only feeble resistance. The two sexes are affected alike. This is shown in the following table :—

| Author. | | Males. | | Females. |
|---|---|---|---|---|
| Conrath | .. | 36 | .. | 31 |
| Demoulin | .. | 48 | .. | 35 |
| Hartmann | .. | 105 | .. | 112 |
| Total .. | .. | 189 | .. | 178 |

*Symptoms.*—There are two different symptom groups which correspond very closely in their incidence with the two pathological varieties of the disease. These are (1) local peritonitis, (2) chronic obstruction associated with a tumour.

*In the entero-peritoneal* form of the disease the attack begins like appendicitis, but is often much less abrupt. With the occurrence of a sudden onset of right iliac pain there generally arises some painful diarrhœa, in which blood and mucous are passed in considerable quantities. The general condition of the patient is good, and there is no rise of temperature. But the indurated mass which has formed in the right iliac fossa, instead of getting smaller, becomes larger and more dense. Then in the course of weeks or months a stercoral abscess is developed, and this by bursting gives rise to fistulæ. These may form in the femoral, inguinal, lumbar or umbilical regions, sometimes opening at the navel itself, or the fistulæ may open into other parts of the bowel. Lung complications commonly precede or accompany this form of the disease, and it is possible that the intestinal condition is often a secondary one.

*In the hyperplastic form* the onset is very insidious, and the patient's general condition may for a long time be that of activity and health. Obstinate constipation, colicky pains, abdominal gurgling and occasional diarrhœa are caused by the slowly progressive stenosis of the bowel, and when this group of symptoms is well established the abdomen exhibits an indurated tumour in the right iliac fossa and the visible peristalsis of hypertrophied bowel, which is so characteristic of chronic obstruction. When my patient (F. L.) was brought to the hospital his abdomen was much distended, and he was in great pain. Through the abdominal wall could be clearly seen many coils of intestine. Very large ones in the position of the transverse and pelvic colon looked like large intestine, and transversely disposed coils represented the small bowel. As the attacks of pain came on the different parts of the intestine could be seen and felt to contract and harden. Deep palpation discovered indurated masses in the right iliac fossa and in epigastric regions, which were proved by laparotomy to be the cæcum and the contracted omentum respectively.

The tumour formed by the indurated cæcum is often higher up than the normal position of this viscus, and this is due to the contraction of the fibrous tissue dragging the caput coli up towards the liver. It, however, often maintains the general outline of the cæcum and colon much more accurately than is the case in malignant disease. The duration of the disease is difficult to estimate because of its insidious onset. My patient had had pain and constipation for a year before obstruction became extreme, and he lived for nearly one year after this was first relieved. Hartmann gives the average duration as being two and a half to three years. Death takes place either from the general cachexia of chronic obstruction or from peritoneal or pulmonary tuberculosis.

*The diagnosis* has to be made in the entero-peritoneal form from appendicitis, actinomycosis, tuberculous lymphadenitis, and other forms of tuberculous abscesses. From appendicitis it is distinguished by the slower onset and the gradually progressive nature of the iliac swelling; also by the greater prominence of blood-stained diarrhœa. Actinomycosis forms a much harder swelling, and one which is more rigidly fixed to the abdominal wall. And when fistulæ form or the mass is opened the characteristic sulphur granules containing the ray fungus escape.

The hyperplastic variety of the disease has to be distinguished from other forms of chronic intestinal obstruction, and especially from malignant growths. This distinction is, however, seldom made before operation, and then often not until the removed tumour has been subjected to microscopical examination. However, this is not of much practical importance, because in both cases treatment by operation is equally demanded at the earliest possible date. In cancer, moreover, the form of the cæcum or colon is obscured and not magnified by the growth, and it often feels more hard and nodular than does a tuberculous mass. The onset of the obstruction is more rapid in cancer, patients often presenting themselves with what appears at first to be primary acute obstruction.

*The treatment.*—Like many other forms of abdominal tuberculosis, this disease is highly amenable to surgical treatment. It is of slow development, remains for a long time definitely localised, and is very late in causing general dissemination. In some cases where the disease is chiefly superficial as a peritoneal affection, a mere exploratory laparotomy may, as is the case in the ascitic variety of tuberculous peritonitis, serve to bring about a permanent cure. Crouzet has related such a case, and Campiche in 19 cases when a simple exploratory operation was performed noted permanent recovery in three.

But when *the entero-peritoneal form* has assumed its usual characters some further steps will be necessary. Usually an exploratory incision is made into the indurated mass and an irregular abscess is encountered, which is walled round by dense adhesions. Only rarely, as in a case narrated by Jopson, is it possible to remove limited foci of disease with a satisfactory recovery. Usually the lumen of the bowel is opened by the ulcerative process of the disease, or by the surgeon's manipulations. This produces a fæcal contamination of the wound which prevents all further operative procedure at the time. A fæcal fistula results, and, as in the case of the boy (F. B.) which I have described, such a fistula is exceedingly difficult to heal. Four operations at different times all failed, and I had at last to resort to a short circuiting operation, after which the closure of the fistula led to permanent healing. And in view of the extensive disease with adhesions which may occupy the whole of the right iliac and lumbar regions, it would seem that in most instances of the entero-peritoneal disease that short circuiting is the best treatment, and it should be resorted to without any undue delay. In the instance mentioned the boy gained three stone in weight within one year, and he is now able to work as an engineer's apprentice. The removal of the primary focus is usually impossible in this variety because of the extensive adhesions, and for the same reason it will be easier to attach the ileum to the pelvic colon than to any other part of the large intestine.

*In the hyperplastic form* the treatment should be conducted on exactly the same lines as is the case in malignant disease. Possibly there may not be the same urgent necessity for a preliminary operation to relieve obstruction in tubercle as in cancer, but Hartmann's own figures show a much higher rate of mortality in the case of primary resections than in the case of anastomosis. In tubercle, as in cancer, there is the same urgency for early exploratory operation, the same advantages derived from a preliminary ileo-colostomy, and the same advisability to remove a good length of intestine with its associated lymph glands. In this, too, even more than in cancer, it may be possible to postpone removal of the main disease until some time after a short circuiting operation; that is to say the fixation by adhesions of a tuberculous mass may be so great when the abdomen is first opened as to prevent the possibility of its removal. Later on, when the fæcal stream has been diverted from the diseased area, it will be smaller and less adherent, and its removal can be then attempted in much worse cases than if it were malignant, because entire removal is not equally necessary.

Hartmann admits that a large number of patients who recover well from operation die eventually from peritoneal and pulmonary tuberculosis, and this is largely favoured by two characteristics of many of the operations which have been performed. In the first place, the recovery after short circuiting operations is not sufficiently often followed up by removal of the focus of disease, and in the second place, the removal of the disease is often too limited. Dobson has removed the termination of the ileum, the cæcum and colon up to the transverse portion, with the peritoneum and lymph glands up to the root of the mesentery. In this case even the highest glands were tuberculous, and such a radical procedure is a good example of what may be successfully done in these cases. In my case (F. L.) the fibrous contraction had advanced so far in the termination of the pelvic colon that I was obliged to make a sacral anus, and so much of the ileum and colon were involved that further operation was impossible.

In some cases where there has been disease limited to the ileo-cæcal region, this has been totally excluded from the intestinal canal by uniting the ileum to the ascending or transverse colon, and cutting through, above and below the disease, fixing the affected bowel into the parietal wound. This usually leads to a permanent fistula, and it would, therefore, be much better to excise the excluded parts at a later date.

Hartmann has collected the following figures relating to the operations for this form of tuberculosis :—

| Number of Cases. | Nature of Operation. | Deaths. | Percentage Mortality. |
|---|---|---|---|
| 9. | Partial resection of cæcum | 1 | 11 |
| 78. | Resections with end to end anastomosis | 19 | 24 |
| 31. | Resections to side to side junction | 5 | 16 |
| 10. | Resections *a deux temps* | 3 | 33 |
| 29. | Ileo-colostomy | 4 | 14 |
| 9. | Unilateral exclusion | 1 | 11 |
| 22. | Bilateral exclusion | 2 | 9 |
| 19. | End to side anastomosis | 3 | 15 |
| 22. | Multiple operations | 8 | 36 |
| 229 | | 46 | 20 |

But the same author points out that the operative mortality has greatly lessened for resections since surgical technique has improved, so that whereas prior to 1900 there were 73 resections with twenty-two deaths (30 per cent.), since 1900 there have been 58 with only seven deaths (12 per cent.).

**Case 1.**—Tuberculosis of the cæcum, rupture, ileo-sigmoid-ostomy. F. B. Boy, aged 19. September 11th, 1907, admitted with severe abdominal pain and collapse. Early in the morning of September 10th he was awakened from sleep by sudden abdominal pain followed by vomiting. The day before he had taken a large quantity of raw fruit. For some time past he had had vague "indigestion" and constipation. His temperature was 101° F., pulse 148, respirations 36. The tongue was dry and furred. The abdomen was distended, rigid and very tender all over, with signs of free fluid in both flanks. Vomiting had continued since the seizure. The bowels had not been opened for four days. Opened after an enema.

*Operation: Peritoneal drainage, removal of the appendix.*—I opened the abdomen over the appendix, being inclined to the diagnosis of appendicitis rather than ruptured gastric ulcer by the fact of the repeated vomiting. A slit was found in the cæcum, 2 cm. long, at the base of the appendix. The latter was removed, and the slit sewn up. Both solid and fluid fæces were free in the peritoneal cavity, and in the light of later observations I think the former may have been washed out of the colon by the enema which was given before the operation. There was a large quantity of thin, stinking fluid in the peritoneum, but no adhesions. The peritoneal cavity was hurriedly mopped out and drained through the appendix wound and through a stab in the right loin. At the end of the operation his condition was desperate, the pulse being 150 and very small. He was transfused per rectum with hot saline fluid, twelve pints slowly running in within twelve hours, and his condition rapidly improved.

September 12th—15th. He became at first drowsy, and then for two days violently delirious, a kind of maniacal attack occurring for ten minutes at a time, and being repeated at intervals of about an hour. Temperature 100–102; pulse 100–118, respirations 24–32.

September 20th—21st. Considerable bronchitis and pleurisy developed at the basis of both lungs, and he had marked cyanosis. The cæcal wound had broken down, and there was a large fæcal fistula.

September 22nd. At 11 p.m a sudden profuse hemorrhage occurred from the the wound, and the house-surgeon held a mass of gauze in it until my arrival. The appendix wound was opened and enlarged downwards, and a bleeding vessel found just over the brim of the pelvis, where the peritoneum appeared to have been erroded by the digestive action of the bacteria and intestinal juices. The bleeding vessel was tied, and the peritoneum sewn over it. The cæcal opening was again closed. He was quite pulseless at the end of this procedure, but revived after a venous infusion—three pints of saline solution.

September 26th. The chest condition has cleared up. The hæmorrhage appears to have done the lungs good. The cæcal fistula has re-opened.

September 29th. A Paul's tube tied into the fistula which lay in the midst of the large gaping wound. I did this because I was afraid that the fluid intestinal contents might again digest the peritoneum. The general condition greatly improved.

On October 9th, 17th and 25th the cæcum was sewn up, but on each occasion broke down again within three days. The attempt to close it up was, therefore, temporarily abandoned, and on two occasions all the fluid fæces coming from the cæcostomy were collected through a Paul's tube together with that passed by the rectum. Professor Walker Hall kindly examined these with the results which I have published elsewhere (*Proc. Roy. Soc. of Med.*, February, 1909).

January 29th, 1908. The boy's condition had improved, and he had gained nearly two stone in weight (November 14th, 6 st. 10 lb.; January 25th, 8 st. 6 lb.); but as it seemed hopeless to close the cæcal fistula by direct operation I decided to do an *ileo-sigmoidostomy.* Iodoform gauze was packed into the cæcum and into the ileum through the fistula, and it was closed by deep fishing-gut stitches, which included the parietal wall. The surface of the abdomen was disinfected and painted over with collodion. A median incision made below the navel. The terminal part of the ileum had a number of fibrous tubercles scattered on its surface, and this gave a clue to the nature of the original disease. The cæcum and ascending colon were buried in adhesions. The pelvic colon was of about the same diameter as the ileum. A lateral anastomosis, two inches long, was made between these parts of the gut. The ileum was completely divided, and the distal end sewn up. The mesentery of the ileum at the anastomosis was sewn to the parietes in order to obliterate the pouch and prevent coils of intestine becoming involved in this. Incision closed without drainage. Made a good recovery.

February 1st, 1908. The fistula had re-opened; the gauze was removed.

From February 2nd, 1908, the bowels have always acted regularly each day without aperients or enemas, the motion being natural in quantity and appearance.

February 28th, 1908. The fistula was closed in four layers, and it healed well and gave no further trouble.

The patient has made an excellent recovery, and is able to work as an engineer's apprentice.

On October 1st, 1908, his weight was 9 st. 6 lb., and both abdominal scars were sound and healthy.

**Case 2.**—Hyperplastic tuberculosis of cæcum and colon extending to the rectum. Colostomy. Ultimately death.

F.L., man, clerk, aged 32. August 25th, 1907. Admitted with abdominal distension and chronic intestinal obstruction.

*History.*—Always well and healthy until six months ago, when he became very constipated, passing only small motions with some pain and straining. This was often succeeded by diarrhœa, the patient going to stool ten to twenty times a day. There was no blood or matter with the motions, but the hard fæces were often covered with slime. For past week there has been no motion at all, and even flatus passes with difficulty, and severe abdominal pain has been present. He began to lose weight twelve months ago, and this has been rapid lately. One of his children, aged 5, died a year ago of tuberculous peritonitis.

*Condition.*—He is thin, and the abdomen is greatly distended by large, visibly contracting coils of intestine in which the colon can be outlined at the periphery and the small intestine in transverse bars at the centre. Over the right iliac fossa and in the epigastric region hard nodular masses can be felt. High up in the rectum was a tight stricture, which only admitted the finger tip after great forcing. The mucous membrane was at this point perfectly normal to sight and touch. A tube could be passed through the rectal stricture, and by this means, and by the aid of oil enemata, he was greatly relieved. But the general abdominal distension remained, and I decided to explore.

September 6th, 1907. *Exploratory laparotomy.* Median incision. The whole of the large intestine, from the cæcum to the rectum, had firm white patches scattered over its surface. These had the appearance of thick leukoplakial patches on the tongue, and they were ½ to 1 cm. in diameter. Here and there one patch was raised and nodular, but there was not any resemblance to ordinary fibrous tubercles. The whole large bowel was enlarged, but there were no adhesions and no free fluid. In the mesentery near the end of the ileum was a hard mass of large lymph glands, which had so contracted the mesentery as to pucker up the intestine for six or seven inches. The whole of the cæcum was densely hard, but it maintained its normal form and position, and the fibrous patches above-mentioned were so closely set on its surface as almost to cover it. The great omentum was puckered up to form a transverse lumpy mass below the transverse colon. It was so dense that it cut like cartilage. Pieces of fibrous nodules and of the omentum were taken for microscopical examination. The floor of the pelvis was hard and indurated by an extraperitoneal exudate, which tightly constricted the pelvic colon and rectum as they passed through it. The wound was closed without drainage. Microscopical examination of all the pieces removed showed nothing but dense fibrous tissue with some round cells. There was nothing to suggest tubercle or new growth.

October 15th. **Sacral Colostomy.** In the left lateral position the back of the sacrum and coccyx were exposed and the latter removed. Dense fibrous tissue, about 2 cm. thick, surrounded the rectum. This was cut away as far as possible, and the pelvic colon reached above the constricting mass. It was fixed to the skin by its posterior edge, and to the lower part of the rectum by its anterior. It was not possible to do an accurate anastomosis.

He left the hospital in November greatly relieved of his distension, the sacral anus acting well.

February, 1908. Readmitted for abdominal distension From November, 1907, to January 7th, 1908, he was in good health and gaining weight, and he could follow his business. For the last month he has become constipated, and the abdomen has been much distended.

*Exploratory operation.*—Median incision. A large quantity of serous but rather viscid fluid removed from the peritoneal cavity. The whole surface of the intestines were covered with closely-set tubercles. The cæcum and the last twelve inches of the ileum were matted together in a hard inextricable mass. The pelvic colon was converted into a large, hard, rigid tube. Closed without drainage. Some of the fluid was collected and sent to the Lister Institute, but its injection into guinea pigs gave a negative result.

The sacral anus led into a much constricted rigid tube which barely admitted the finger.

May, 1908.—I saw him at his own house. He was in constant and severe pain. There was a moderate amount of free fluid in the abdomen, which was occupied by many hard masses which seemed to fill the whole right iliac lumbar hypochondriac and epigastric regions. Vomiting was frequent, and hardly anything passed from the bowel. He died in June, 1908, but no autopsy was possible.

### REFERENCES.

Courath, *Beitr. z klin. Chir.*, 1898, xxi.
Hartmann, *Brit. M. J.*, 1907, i, 849.
Dobson, *Lancet*, 1908, i, 149.
Cumston, *Ann. Surg.*, 1907, xlvi, 765.
Crouzet, Tuberculose caecale. Lyon, 1905.
Campiche, *Deutsch. zeit f. Chir.*, 1905, lxxx, 495.
Demoulin, *Bull. et Mem. Soc. de Chir.*, 1905, xxxi, 636.
Guinon and Pater, *Bull. Soc. de Pediat.*, 1906, viii, 163.
Jopson, *Ann. Surg.*, 1908, xlvii., 635.
Matas, *New Orleans M. and S. J.*, 1907—8, lx, 840.
Cornil, *Bull. et Mem. Soc. Anat. de Paris*, 1907, lxxxii, 505.
Groves and Walker Hall, *Proc. Roy. Soc. Med.*, Feb., 1909.

